# Older Adults with Dementia: Association of Prayer with Neuropsychiatric Symptoms, Cognitive Function, and Sleep Disturbances

**DOI:** 10.3390/rel13100973

**Published:** 2022-10-17

**Authors:** Katherine Carroll Britt, Kathy C. Richards, Gayle Acton, Jill Hamilton, Kavita Radhakrishnan

**Affiliations:** 1 School of Nursing, University of Pennsylvania, Philadelphia, PA 19104, USA; 2 School of Nursing, The University of Texas at Austin, Austin, TX 78712, USA; 3 Nell Hodgson Woodruff School of Nursing, Emory University, Atlanta, GA 30322, USA

**Keywords:** religion, spirituality, coping, Alzheimer’s, supplication, illness

## Abstract

Protective factors that slow dementia progression and improve quality of life are needed. Neuropsychiatric symptoms (NPS), cognitive decline, and sleep disturbances are commonly found in dementia, indicate progression, and increase caregiver distress. The purpose of this study was to examine the association of private prayer with NPS, cognitive function, and sleep disturbances in older adults with dementia. We analyzed data from the Health and Retirement Study in 2000, 2006, and 2008 and Aging, Demographics, and Memory Sub study in 2001–2003, 2006–2007, and 2008–2009 among 40 older adults (age 70–100 years, mean age = 84.67, 29 females and 11 males, 73.9% non-Hispanic White, and 19.2% Non-Hispanic Black, and 3% Hispanic, cognitive function = 1.169 indicating mild cognitive impairment) using correlational analysis. The results indicated that increased frequency of private prayer was significantly associated with lower NPS, better cognitive function, and lower sleep disturbances. In total, 100% of Non-Hispanic Black and Hispanic participants reported praying at least once per week. Findings could be due to use of cognitive processes used in prayer during supplication, requesting aid, and through communication with the divine, reducing loneliness. Longitudinal studies including historically underrepresented populations are needed to examine these associations over time.

## 1. Introduction

Dementia is one of the costliest conditions to society and a public health priority (World Health Organization 2017). An increasingly prevalent terminal illness, dementia does not presently have a cure and it cannot be prevented. In 2015, the United States spent over $800 billion on dementia care and it is estimated that by 2050, 152 million people will have dementia worldwide (World Health Organization 2017). Alzheimer’s disease is the most common cause of dementia and can affect thinking, memory, independence, learning, speaking, understanding speech, and communication (Alzheimer’s Association 2021).

As the disease progresses, persons with dementia often rely on others for care and support of their needs. Neuropsychiatric symptoms (NPS), cognitive decline, and sleep disturbances are commonly found in dementia and indicate disease progression (Okura et al. 2010; Santacruz Escudero et al. 2019). With symptoms such as aggression, apathy, wandering, anxiety, and depression, NPS in persons with dementia can be difficult to manage, prompt earlier institutionalization, and increase caregiver burden (Mehta et al. 2003; Scarmeas et al. 2005; Tsunoda et al. 2020). Declining cognitive function prompts increased reliance on caregivers for daily needs and activities. Manifestations of sleep disturbances include frequent nighttime awakenings and increased daytime napping which in turn may affect caregiver sleep as well. With increasing methods of early identification of individuals at greater risk of dementia through precision health and with the increasing dementia burden, identifying ways to slow disease progression is warranted, especially as up to 35% of risk factors are modifiable (Livingston et al. 2020).

### 1.1. Religious and Spiritual Practices

The utilization of religion and spirituality in times of uncertainty, stress, and illness is well documented (Balboni et al. 2022) and as adults age, religion and spirituality may become more important (Katsuno 2003; Tornstam 1996; Thunstrom and Noy 2022). Among older adults, religious and spiritual practices may play a protective role in cognitive decline as reported by a systematic review of several studies by Hosseini et al. (2019). Additionally, frequency of prayer in midlife is associated with lower risk of preclinical dementia, adjusting for age and education, among Arabic women (Inzelberg et al. 2013). However, few studies have examined religious and spiritual practices among persons with dementia (Britt et al. 2022a). For persons with Alzheimer’s disease, McGee et al. (2013) report negative religious coping is associated with fewer behavioral symptoms in persons with Alzheimer’s disease in the U.S. in a cross-sectional study (*N* = 28). These studies suggest religious and spiritual practices may be protective, but more studies are needed (Britt et al. 2022a), especially upon dementia progression to slow the dementia burden and to improve quality of life.

### 1.2. Theoretical Framework

**Figure 1.**
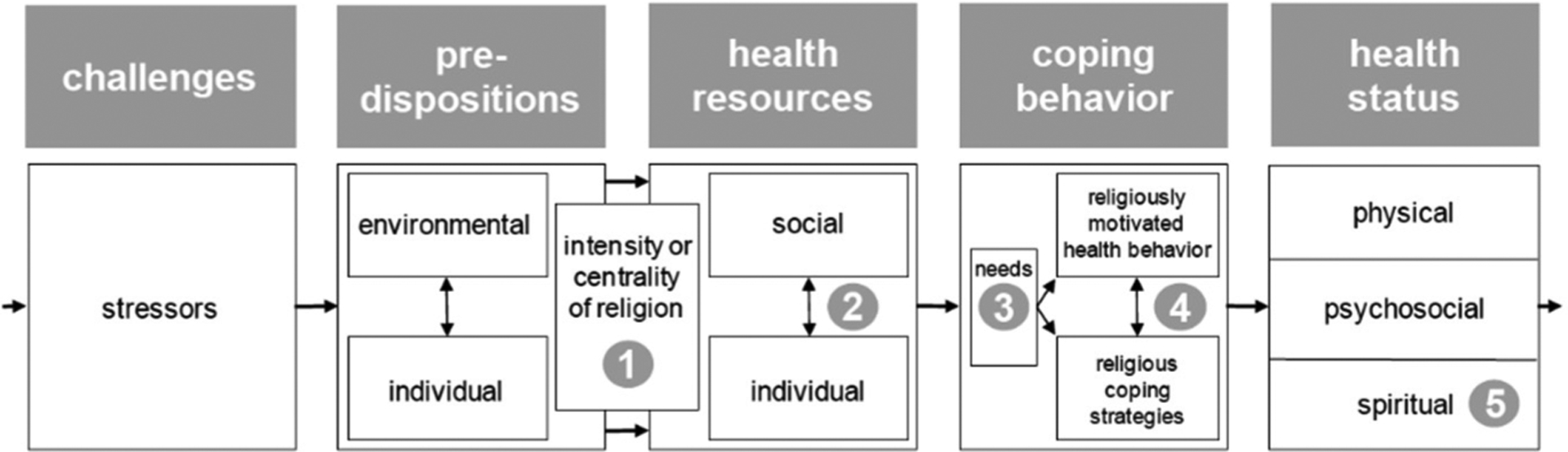
The Vulnerability-Stress Model incorporating Religiosity/Spirituality (VSM-RS) which includes (1) centrality of religion, (2) R/S resources, (3) spiritual needs, (4) religious coping, and (5) spiritual well-being.

This study was informed by the vulnerability-stress model incorporating religiosity/spirituality (VSM-RS) which served as a conceptual guide (Zwingmann et al. 2011) (see Figure 1). This VSM-RS framework displays a heuristic, linear model to project a pathway from a stressor to health incorporating factors of predispositions, health resources, and coping behaviors. Positing health outcomes are the result of a combination of these factors in response to a stressor, Zwingmann and colleagues (2011) suggest five dimensions of religiosity/spirituality along the linear path which include (1) centrality of religion/spirituality (i.e., religiosity, spirituality, religiousness), (2) religious and spiritual (R/S) resources, (3) religious coping, (4) spiritual needs, and (5) health outcomes using spiritual well-being. Based on established theories (i.e., diathesis-stress model, transactional theory of stress and coping), it posits individuals embrace religion and spirituality found in individual and environmental predispositions, coping behaviors, and resources in response to a stressor perceived to exceed a threshold (Ingram and Price 2001; Lazarus and Folkman 1984; Zuckerman 2009). Using the model as a guide, the present study concentrated on the concepts identified in the framework: (1) centrality of religion/spirituality conceptualized as private R/S activities (i.e., private prayer) and individual predispositions (e.g., race/ethnicity, gender), and (2) health outcomes, conceptualized as NPS, cognitive function, and sleep disturbances. The present study is strengthened by the model’s framework providing clear boundaries for religion, spirituality, and health dimensions and is based on R/S measures utilized in health research (Steinhauser and Balboni 2017).

### 1.3. The Present Study Purpose

The purpose of this study was to examine associations between private prayer and NPS, cognitive function, and sleep disturbances among older adults with dementia.

## 2. Methods

### 2.1. Study Design and Population

To examine these associations, we conducted a secondary analysis of publicly available data utilizing The Health and Retirement Study (HRS) and sub study, Aging, Demographics, and Memory study (ADAMS). The HRS consists of a nationally representative sample of adults aged 50 and above across the U.S. collecting data every two years beginning in 1992 to examine cognition, demographics, behavioral and social science factors and health. ADAMs is a sub study of the HRS sampling U.S. older adults aged 70 years and above every 1.5–2 years beginning in 2001, and was designed to examine health, risk factors, costs, and prevalence of dementia and cognitive impairment. A participant’s record was dropped if more than 5% of responses were missing from the record.

### 2.2. Procedures

For persons with all-cause dementia, data were examined for frequency of private prayer on NPS, cognitive function, and sleep disturbances. Data were used from the RAND HRS Longitudinal and Fat Files in 2000, 2006, and 2008, and in ADAMS waves 2001–2003, 2006–2007, and 2008–2009. Data were obtained from participants with all-cause dementia who completed the HRS survey for private prayer and covariates in HRS and in ADAMS for NPS, global cognition, and sleep disturbances.

### 2.3. Measures

In ADAMS, a dementia-trained nurse and neuropsychological technician conducted in-person assessments in the participant’s residential setting with presence of a familiar surrogate required. A neuropsychological test battery was conducted, a chronological history was taken (medical, psychiatric, cognitive function, family history, behavioral symptoms), as well as prior neuroimaging and laboratory test results collected from participants’ physicians (Langa et al. 2005). Final diagnosis was established by consensus panel of clinical experts (Gure et al. 2010) based on the DSM-III-R (American Psychiatric Association 1987) and DSM-IV (American Psychiatric Association 1994). HRS weights were created for sampling of participants and nursing home status.

NPS measures used a structured caregiver interview using the Neuropsychiatric Inventory (NPI), a widely accepted measure utilized in cognitive impairment to assess frequency, severity, and presence of behavioral and psychiatric expressions across 10 dimensions: aberrant motor behaviors, apathy, agitation/aggression, anxiety, delusions, depression, disinhibition, elation, hallucinations, and irritability (Cummings et al. 1994; Kaufer et al. 1998). An additional dimension was added in the ADAMs study, sleep disturbances. For NPI scoring, absence of a neuropsychiatric symptom was coded as 0; if the symptom was present, informants reported frequency by severity of symptoms with total scores calculated by multiplying symptom frequency and severity. A score of 4 or more was deemed clinically and meaningfully significant (Schneider et al. 2001). Scale validity and strong reliability have been reported in other studies (Ismail et al. 2016). Captured by three items with yes/no response, sleep disturbance scores were summed by these three items to create one total score composite with range from 0–3. Higher scores indicated higher degree of disturbed sleep collected across these three items: (1) (a) Do you have problems falling asleep? (b) Do you wake frequently? and (c) Do you have trouble waking too early? (Jelicic et al. 2002; Moon et al. 2017). To retain partial responses for sleep disturbance, responses across the 3 items marked “98” (don’t know) were marked as 0.

Cognition and functional performance were rated by the Clinical Dementia Rating (CDR) based on information provided by participants and the surrogate during evaluation (Hughes et al. 1982; Morris 1993). Scored across a 5-point scale quantifying severity of cognitive impairment with ranges: normal (0), very mild dementia (0.5), mild dementia (1), moderate dementia (2), and severe (3) (Gure et al. 2010; Morris 1993), this cognition score was created based on evaluation across six domains: memory, orientation, problem solving, community affairs, hobbies, and personal care.

Private spiritual practice was measured as frequency of private prayer. For HRS 2000, participants were asked, “Do you ever pray privately in places other than at church or synagogue?” A dichotomous response (yes/no) was recorded; for those answering “yes”, a follow up question was asked, “How often do you pray privately?” Five response scores range from less than once per month (0) to daily (5). For HRS 2006 and 2008, “How often do you pray privately in places other than at church or synagogue?” Five responses range from (1) more than once per day to (8) never in HRS 2006, and from (1) daily to (6) not in the last month in HRS 2008. Due to smaller sample size and response frequencies, responses were recoded into 3 categories across all data: none/not at all (0), less than once per week (1), and once per week or more (2). Selected based on workgroup recommendation for HRS (Levin 2003), this item for private prayer is widely applicable across the U.S. serving as a sensitive indicator of religious activity (Levin et al. 1994).

Demographics included age (years), sex (male = 0, female = 1), education (years), social contact (“frequency of getting together with people” range from (1) day to (6) almost never), and race/ethnicity (0 = non-Hispanic White, 1 = non-Hispanic Black, 2 = non-Hispanic Other, 3 = Hispanic).

### 2.4. Statistical Analysis

Descriptive statistics were calculated for age, marital status, sex, education, social contact, race/ethnicity, living location (i.e., nursing home, community), religious preference, private prayer, and importance of religion. To examine the relationship between private prayer and NPS, cognitive function, and sleep disturbance, we calculated a bootstrapped Spearman’s Rho correlation using IBM SPSS Statistics (Version 25). To account for the complex sampling design (stratification, clustering, nonresponse) of both the HRS and ADAMS, all analyses were weighted and adjusted (Heeringa et al. 2009).

Data were analyzed for missing data, errors, and presence of multicollinearity. Frequency distribution of all categorical variables and mean, standard deviation, and percentiles of all continuous variables were obtained.

## 3. Results

The sample included 40 older adults (74.9% females) aged 73–100 (*M* = 84.67, sd = 5.16) (see Table 1). Most were non-Hispanic White participants (73.9%), living in the community (93.2%), widowed (56.2%), Protestant (66.6%), and mean education was 9.84 years. The remaining participants identified as Catholic (32.2%) or other (1.2%) for religious preference. Participants reported religion as very important (69.5%) with 91% praying at least once or more per week. Around 30.2% reported almost never having any social contact, 30.1% having social contact every year, followed by 22.2% reporting daily social contact. Cognitive function was 1.169 (sd 0.516), indicating mild cognitive impairment, mean score of NPS 5.22 (sd 7.41) was identified as clinically significant, and mean sleep disturbances 0.79 (sd 1.078) were mild on 0–9 score.

Approximately 100% of non-Hispanic Black and Hispanic participants both reported praying at least once a week compared to 87.8% of non-Hispanic White participants (see Table 2).

Across sex differences, 89.1% of females reported praying daily compared to 96.4% males while 67.5% females compared to 75.6% males reported religion as very important (see Table 3).

Data did not meet the assumptions of regression analysis due to heteroscedasticity, nonnormal distribution, and nonlinearity as assessed with regression assumptions, therefore, a nonparametric correlation analysis was conducted using bootstrapped Spearman’s correlation for all 3 outcomes: NPS, CDR, and sleep disturbance.

Private prayer was significantly associated with NPS (r_s_ (97) = −0.358, 95% CI [−0.363, −0.353], *p* < 0.01), cognitive function (r_s_ (97) = −0.383, 95% CI [−0.388, −0.378], *p* < 0.01), and sleep disturbances (r_s_ (97) = −0.147 95% CI [−0.153, −0.141], *p* < 0.01) (see Table 4).

## 4. Discussion

Our findings indicate increased frequency of private prayer was associated with lower NPS, better cognitive function, and lower sleep disturbances. These are similar to other previously reported research conducted in Italy among persons with mild and moderate Alzheimer’s disease (*N* = 64) suggesting higher religiosity (i.e., frequency of religious activity participation) and spirituality (i.e., defined as one’s personal attitude towards Christianity) are associated with slower cognitive and behavioral decline over 12 months (Coin et al. 2010). The type of prayer in the present study was not differentiated but in a recent qualitative study among caregivers and their loved ones with dementia, supplication prayer was utilized by dementia dyads to request aid for friends and loved ones experiencing illness or difficult life circumstances during social distancing; it was spoken of as a positive action an individual with limited functional ability can utilize to help others (Britt et al. 2022b). Among African American adults with history of life-threatening illness, prayer was used for requesting strength to endure, for healing, to give thanks, and ask for protection (Hamilton et al. 2019); their prayers helped them feel connected to God, to self, and to others, supporting their spirituality. Our studies’ findings of lower behavioral expressions, better cognitive function, and lower sleep disturbances could be due to the use of cognitive processes used in prayer to seek guidance for making decisions, to request aid when overwhelmed, in prompting positive psychological emotions such as hope, and by reducing loneliness through communication with the divine.

Though empirical evidence supporting positive effects of private prayer in health outcomes are limited, individuals may find hope in utilizing prayer, believing that it helps them (Masters and Spielmans 2007). According to Laird et al. (2004), there are five types of prayer an individual may utilize: adoration, confession, thanksgiving, supplication, and reception. Adoration prayer refers to praising and worshipping God without referencing needs or desires while confession prayer refers to admitting negative behavior and thought, seeking forgiveness (Laird et al. 2004). Thanksgiving prayer is expressing gratitude for positive life circumstances, supplication prayer refers to seeking intervention from God in personal life events or on behalf of others, and reception prayer is passively awaiting divine attainment of wisdom, guidance, and understanding (Laird et al. 2004). Adoration prayer is associated with meaning in life and optimism and negatively associated with depressive symptoms (Whittington and Scher 2010; Perez et al. 2011). It is a form of submission to a higher power and worship. Confession predicts poorer well-being, self-esteem, and optimism thus utilized when individuals acknowledge misdoings, shortcomings, requesting forgiveness (Laird et al. 2004; Whittington and Scher 2010) resulting in more positive psychological and physiological outcomes (Martinez-Pilkington 2007; Vitz and Mango 1997). A predictor of better wellbeing, self-esteem, optimism (Whittington and Scher 2010) and lower depression (Perez et al. 2011), thanksgiving prayer is utilized to express gratitude for perceived provisions and positive life circumstances. Supplication prayer is negatively associated with well-being and life satisfaction and represents requests or petition to God for help for self or others. Reception prayer represents contemplation—waiting for guidance or understanding from the divine. It is associated with better self-esteem, meaning in life, and optimism (Whittington and Scher 2010) and negatively associated with depressive symptoms (Perez et al. 2011). The current sample consists of a majority of Protestant (66.6%) and Catholic (32.2%) older participants in the U.S. possibly indicating a narrower, institutionalized-ritual interpretation of prayer. Though a diverse, cross-cultural phenomenon, broader concepts of prayer are utilized across the world including centering meditation as a contemplative practice (Dorais and Gutierrez 2021), deep breathing through prayer therapy (Sadeghimoghaddam et al. 2019), and music in prayer (Koen 2005).

Perhaps prayer provides some sense of control in debilitating illness. Persons with dementia may find peace in prayer practice, thus we see lower apathy, aggression, depression, and agitation. In another study among U.S. adults with acute coronary syndrome, 59.3% reported praying for their health with 85% reported finding strength and comfort in their spiritual practice (Abu et al. 2019). Among U.S. adults with cancer, prayer was negatively associated with depressive symptoms across many types of prayer: supplication, thanksgiving, adoration, and reception (Perez et al. 2011). In India, 90% of adult cancer patients (N = 300) reported prayer made them feel better and higher spiritual distress was also associated with higher pain scores (Bhatnagar et al. 2017). However, in a palliative care clinic in Brazil, individual prayer activity among adults with advanced cancer (N = 221) was not associated with quality of life (Paiva et al. 2014).

Utilizing religion and spirituality to cope may reduce stress and anxiety by providing resources such as beliefs, practices, and rituals, providing a greater sense of meaning and purpose in the midst of challenging life circumstances (Koenig 2012). Though the pathway between these is unclear, it appears religious and spiritual practices promote social interaction and support, increase positive psychological emotions, prompt cognitive stimulation, reduce stress and anxiety, and support healthy lifestyle behaviors (Chen et al. 2021; Koenig 2012). It is possible that psychological factors influence biological means through interactions with the central nervous system and immune systems (Kiecolt-Glaser et al. 2002). With prayer, those who are facing illness may practice petitioning, a form of supplication, requesting control of symptoms or to return to a healthy state (McCaffrey et al. 2004; Ross et al. 2008). For persons with dementia, a decline in independence and communication may prompt individuals to pray—an unlimited and nonpharmacological action that promotes hope and is associated with greater well-being, greater purpose in life, greater satisfaction, and optimism (Anderson and Nunnelley 2016; Laird et al. 2004; Olver and Dutney 2012; Tosta 2004). Thunstrom and Noy (2022) report individuals who positively value prayer believe prayer will give them comfort and support and will be answered by God to improve their health and wealth.

As 69.5% of our sample reported religion to be very important, persons diagnosed with dementia utilized spiritual practice as a coping activity. Similar research reports older adults are more religious than younger populations with religion and spirituality becoming more important with age (Bengtson et al. 2015; Pew Research Center 2018). Prayer is valued across faiths and cultures (Masters and Spielmans 2007) and can be used as a strategy to cope with adverse life circumstances. As seen in our findings, importance of religion and frequency of prayer was higher among historically underrepresented participants and males. Religion and spirituality are culturally salient factors among racial and ethnic minority communities reporting higher rates of religious and spiritual views and behaviors compared to White counterparts (Chatters et al. 2013). Socially marginalized populations report using religious and spiritual practices for psychological support and coping (Chatters et al. 2013). Around 62% of U.S. adults report utilizing some form of alternative medicine of which prayer for themselves (43%) and prayer for others (24.4%) were two of the most commonly reported alternative therapies to medical treatment (Barnes et al. 2002). McGee et al. (2013) in a cross-sectional study of adults with mild Alzheimer’s disease (*N* = 28), 90.4% practiced private prayer some or most days and 95.7% found relationship with the transcendent to be very important. These findings support the importance for spiritual activity and need for increased spiritual practice interventions created to support the spiritual activities of persons with dementia to continue cognitive exercise, improve well-being, and to provide hope and comfort through their disease trajectory.

## 5. Conclusions

A key component of successful aging, lifestyle factors such as religious and spiritual practices may reduce the risk of cognitive impairment and improve mental and physical health (Balboni et al. 2022; Coelho-Junior et al. 2022; Paillard-Borg et al. 2009) but more research is needed examining associations with dementia progression using a larger sample size. Most published studies have looked at prayer among adults with advanced illness and dementia caregivers but there is a large gap among persons with dementia. More studies are needed examining associations over time for religious and spiritual practices among diverse populations. Clinical trials should be created and designed around personal preferences and cultural sensitivity for persons with dementia upholding dignity and supporting person-centered care.

## Figures and Tables

**Table 1.** Description of Dementia Participants (*N* = 40).

Demographics	Mean (SD), Range
Age	84.67 (5.159), 73–100
Education	9.84 (4.027), 0–16
Income	20,602.00 (18,440.31), 5280–97,020
Cognitive Rating	1.169 (0.5164), 0.5–3.0
NPS scores	5.22 (7.413), 0–35
Sleep Disturbance	0.79 (1.078), 0–3
	*Unweighted Count (N* = 40) * (%)
Race/Ethnicity	
Non-Hispanic White	26 (73.9)
Non-Hispanic Black	11 (19.2)
Non-Hispanic Other	0 (0)
Hispanic	3 (6.9)
Gender	
Females	29 (74.9)
Males	11 (25.1)
Proxy	
Yes	1 (0.7)
No	39 (99.3)
Living Arrangements	
Community	38 (93.2)
Nursing Home	3 (6.8)
Marital Status	
Single	0 (0)
Married, partnered	14 (38.7)
Divorced, separated	3 (5.1)
Widowed	23 (56.2)
Religious Preference	
Protestant	27 (66.6)
Catholic	12 (32.2)
Jewish	0 (0)
None/no preference	0 (0)
Other	1 (1.2)
Importance of Religion	
Not too important	2 (6.8)
Somewhat important	9 (23.6)
Very important	29 (69.5)
Private Prayer	
Never or not in the last month	3 (9)
At least once a month	0 (0)
At least once a week	33 (91)
Frequency Social Contact	
Almost never	12 (30.2)
Year	13 (30.1)
Month	1 (2.8)
Every 2 weeks	5 (12)
Week	1 (2.8)
Day	8 (22.2)

Note.

* Table contains raw counts and survey-weighted: means, standard deviations, median, ranges, and percentages; therefore, percentages may not sum to 100.

Cognitive Rating: higher number indicates more impairment. NPS = neuropsychiatric symptoms; CDR = clinical dementia rating; SD = standard deviation.

**Table 2.** Frequencies across race/ethnicity.

Variables	In Dementia (*N* = 40)			
	Non-Hispanic White	Non-Hispanic Black	Non-Hispanic Other	Hispanic
	*N*ˆ (%)	*N*ˆ (%)	*N*ˆ (%)	*N*ˆ (%)
Importance of Religion				
Not too important	2 (9.3)	0	0	0
Somewhat important	9 (31.9)	0	0	0
Very important	15 (58.8)	11 (100)	0	3 (100)
Frequency of Private Prayer				
Less than once a month	3 (12.2)	0	0	0
At least once a month	0	0	0	0
At least once a week	23 (87.8)	11 (100)	0	3 (100)
	Mean (SD)	Mean (SD)	Mean (SD)	Mean (SD)
NPS	5.24 (8.655)	4.21 (9.632)	0	0.67 (0.469)
Cognitive Rating	1.232 (0.5735)	0.955 (0.301)	0	1.00 (0.000)
Sleep Disturbance	0.66 (0.962)	0.75 (0.935)	0	1.43 (1.285)

Notes.

ˆ Table contains raw counts and survey-weighted: means, standard deviations, median, ranges, and percentages; therefore, percentages may not sum to 100. Cognitive Rating: higher number indicates more impairment.

**Table 3.** Frequencies of Females and Males.

Variables	In Dementia (*N* = 40)			
	Females		Males	
	*N*ˆ (%)	Mean (SD)	*N*ˆ (%)	Mean (SD)
Importance of Religion				
Not too important	1 (5)		1 (12.4)	
Somewhat important	7 (27.5)		2 (12.1)	
Very important	21 (67.5)		8 (75.6)	
Frequency of Private Prayer				
Less than once a month	2 (10.8)		1 (3.6)	
At least once a month	0		0	
At least once a week	27 (89.1)		10 (96.4)	
NPS		5.66 (10.08)		3.44 (5.260)
Cognitive Rating		1.246 (0.5548)		1.029 (0.455)
Sleep Disturbance		0.82 (1.104)		0.50 (0.660)

Notes.

ˆ Table contains raw counts and survey-weighted: means, standard deviations, median, ranges, and percentages; therefore, percentages may not sum to 100.

Cognitive Rating: higher number indicates more impairment. SD = standard deviations; NPS = neuropsychiatric symptoms.

**Table 4.** Bootstrapped Spearman’s Rho Correlation Analysis Results.

Variable	NPS	Cognitive Rating	Sleep Disturbance
	*r* (CI)	*r* (CI)	*r* (CI)
Private Prayer	−0.358 (−0.363, −0.353)*	−0.383 (−0.388, −0.378)*	−0.147 (−0.153, −0.141)*

Note. Cognitive Rating: higher number indicates more impairment. *r* = correlation; CI = Confidence Interval; NPS = neuropsychiatric symptoms; CDR = clinical dementia rating.

* *p* < 0.01.

## Data Availability

The data utilized in this study are publicly available at https://hrs.isr.umich.edu (accessed on 2 November 2021).
